# The provision of and need for social support among adult and pediatric patients with tuberculosis in Lima, Peru: a qualitative study

**DOI:** 10.1186/1472-6963-13-290

**Published:** 2013-07-31

**Authors:** Valerie A Paz-Soldán, Rebecca E Alban, Christy D Jones, Richard A Oberhelman

**Affiliations:** 1 Tulane University School of Public Health and Tropical Medicine, 1440 Canal Street, New Orleans, LA 70112, USA; 2 Universidad Peruana Cayetano Heredia, Facultad de Salud Pública y Administración, Avenida Honorio Delgado 430, San Martin de Porres, Lima, Peru

**Keywords:** Social support, Tuberculosis, Peru, Psychosocial

## Abstract

**Background:**

Tuberculosis (TB) remains a significant public health problem in Peru, causing an estimated 35,000 new cases each year, 6.7% of whom are co-infected with HIV. Social support mechanisms are key in influencing health-seeking behavior, adherence, and overall patient wellbeing in clinical settings. We examine the types of social support received by TB patients and parents of pediatric patients in peri-urban Lima, Peru, to understand its role in patients’ psychosocial wellbeing during treatment.

**Methods:**

Semi-structured interviews were conducted between August 2004 and May 2005 among 43 individuals: 19 adults with TB, 8 adults with TB/HIV, 13 parents of pediatric TB patients, and 3 parents of pediatric TB/HIV patients.

**Results:**

Patients described the need for psychosocial support to mitigate the difficulty of continually going to the clinic to take medications, tending to other family or professional responsibilities while on treatment, and confronting stigma and social isolation within their community. Family members most often contributed to meeting these psychosocial needs, and were also crucial in providing economic support to patients faced with burdensome medical expenses or who were forced to leave their jobs due to being on treatment. Most healthcare personnel were described as key providers of emotional support and encouragement for patients to successfully adhere to treatment, however there were a select few doctors whose “scare tactics” seemingly discouraged patient adherence. During the treatment process, patients described being more socially withdrawn as a result of feeling fatigued from their medications, however most participants also described forming new mutually supportive friendships among their fellow patients.

**Conclusions:**

Despite the general reluctance of patients to disclose their disease status, patients received a significant amount of psychosocial support from both family members to whom they disclosed, and from positive interactions with healthcare providers. High levels of depression were reported, with many patients voicing need for improved and more frequent psychological interventions. To improve the Peru TB program, participants suggested extending educational opportunities to patients’ families and the wider community, increasing the existing amount of nutritional support, and programmatic provision of vocational activities to increase economic opportunities.

## Background

Worldwide, tuberculosis (TB) continues to be a significant public health problem: there are an approximate 140 new cases per 100,000 population globally, and in 2009 alone, approximately 1.7 million deaths were due to TB throughout the world [1]. TB treatment programs, such as internationally recommended direct observed treatment short course (DOTS), have demonstrated increased adherence to medication and successful treatment outcomes [2]_._ However some skepticism does exist among experts who are not convinced that there is sufficient data to prove its effectiveness [3]. Factors associated with poverty, such as malnutrition, overcrowding, poor ventilation, unemployment, and lack of access to healthcare prevent greater control of the disease in many developing countries [4-8].

Although the population of Peru is only 3 percent of the Americas, it bears 12 percent of the region’s TB cases [9]. In 2007, Peru had the largest number of new TB cases in South America and the highest reported number of new and relapse cases of TB in South America per 100,000 people, with approximately 35,000 cases in total [10]. In 2006, there were 18,855 cases of TB in Peru’s capital city of Lima alone [11]. Moreover, a significant portion of TB patients is co-infected with HIV: in Peru, in 2007, 2400 of the 35,000 new cases of TB were among patients also infected with HIV [10]. Although Peru has an exemplary TB program [12] and an HIV prevalence rate of less than .5%, rates of multidrug-resistant tuberculosis (MDR-TB) have continued to increase in recent years, making Peru one of the top eight MDR-TB burdened countries in the world [13,14]. Cases of tuberculosis, MDR-TB, and most recently extremely drug resistant tuberculosis (XDR-TB) are highly concentrated in Lima compared to the rest of Peru; from 1997 to 2007, 87% of MDR-TB cases in Peru occurred within the capital and its surrounding areas [14,15]. In 1990, Peru’s Ministry of Health (MINSA) integrated DOTs into the structure of the National TB Program, requiring that patients be observed taking their TB medications by a healthcare professional at their respective health center. DOTS coverage currently extends to 100% of patients enrolled in Peru’s National TB program [16], including all participants in this study. Peru’s National TB Program also supplies food aid and psychological consultations to patients. Nonetheless, greater control of TB was hindered by the increase in HIV/AIDS cases with TB co-infection and the increased cases of multi-drug resistant TB (MDR-TB) [2,8].

Social support has been categorized in various ways, but overall denotes social interactions that “lead the subject to believe that he is cared for, loved, esteemed and a member of a network of mutual obligations” [17]. There is evidence that social support is key in influencing health-seeking behaviors, treatment adherence and health outcomes [18-20]. However, there are limited studies that examine the role of social support specifically with regards to patients with TB, and that furthermore outline the particular components of social support that are helpful or harmful to such patients. This study aims to discuss these matters within the sociocultural context of a peri-urban sub-section of Lima, Peru. Several studies in developing countries describe that improved quality of care by health providers and increased support from family during TB treatment can lead to increased adherence to medication and an improved quality of life for TB patients [4,5,21-24]. These studies emphasized the importance of emotional support from health providers and supportive networks, such as neighbors and family, throughout the duration of TB treatment [4,21,22]. Furthermore, although studies in both developing and developed countries have shown that social support can act as a buffer to stress for patients and help in their recovery, certain interactions can also result in feelings of isolation and distance within relationships [25,26].

This study seeks to explore, qualitatively and from the patient’s perspective, the role of social support during individuals’ TB treatment, including the voices of those with HIV co-infection and parents of pediatric patients. Specifically, the primary objectives of this study are to: 1) explore the types of social support that TB and TB/HIV co-infected patients in Lima experience during TB treatment, with a particular focus on its role in the patient’s psychosocial wellbeing, and possible implications on treatment adherence; and 2) explore, from the patients’ perspective, ways in which the Peru TB program could be improved in terms of providing social support and other non-clinical services. Because we were interested in learning about TB patients’ experience of social support and psychosocial wellbeing during treatment, and hearing about it in their own words, we chose to explore this topic in a qualitative manner, using open-ended semi-structured questions. These findings can guide the development of interventions for patients’ families, caregivers, and health providers, which ultimately could lead to improved patient wellbeing, and in turn, more successful treatment outcomes.

## Methods

Using semi-structured interview guides, 43 in-depth interviews were conducted in 2004 and 2005 with adults in the process of receiving DOTS treatment for TB (n = 27) and parents of children receiving treatment for TB (n = 16); of these individuals, there was a sub-group that were co-infected with HIV (adults, n = 8; children, n = 3). Four similar interview guides were created, one for each sub-population (TB, TB/HIV, parent of pediatric TB, parent of pediatric TB/HIV), but all including the same topics: description of the process of being diagnosed; their knowledge of TB (and HIV for TB/HIV cases); their feelings throughout the process of learning about and accepting the TB diagnosis; the types of interactions they had or would have liked to have with health care providers, family and friends associated to their TB treatment and recovery; how their interactions with loved ones changed during the course of their treatment; what helped them continue their treatment for TB; and what suggestions they had for obtaining the type of support they would have liked from family, friends, and providers. The interviews took approximately one to one and a half hours.

### Setting

This study took place in Lima, Peru (estimated population 8,350,000) [27]. Lima is divided into five main health regions: participants were recruited from 5 health centers and the regional hospital in the southern region of the city (DISA-South estimated population 2,105,296). Parents of TB and TB/HIV patients were recruited from the same health centers, as well as from the Instituto Nacional de Salud del Niño (the Peruvian Children’s Hospital). All TB patients were enrolled in DOTS therapy, which required that they be observed taking their TB medications by a healthcare professional at their respective health center.

### Sampling, recruitment, and sample size

Purposive sampling was used to select people that would be able to provide specific knowledge about social support associated with diagnosis and treatment for TB in Lima [28]. Our sampling criteria included adults, both male and female, who were in current treatment for TB – whether “only” with TB or co-infected with HIV – or parent of a child in treatment for TB – again, whether only with TB or co-infected with HIV. We hypothesized that people with co-infection might be dealing with additional emotional or stigma issues compared to those with TB. We also hypothesized that this group might have different types of resources available to them, and therefore, wanted to hear about the experience of this particular sub-group. We also focused on individuals who were currently in treatment, and who had been in treatment for at least several weeks to give us a broader perspective on their emotions and social support at different phases of the treatment process. Two psychologists working in the national TB program in clinics in DISA-North were trained for two weeks by the PI (VPS), a Peruvian social scientist, to conduct the interviews in the above-mentioned health facilities in DISA-South. Training consisted of reviewing the goals of the study, practicing the interviews with the PI or with the PI observing and providing feedback, and finally, the PI was present at approximately eight of the initial interviews of each assistant, and would listen to the tapes of the interviews as they arrived. Participants were recruited through clinician gatekeepers. Medical personnel briefly described the study to adult patients and parents of pediatric patients who came in for TB treatment. Those interested in participating were then introduced to one of the research assistants, who described the study in greater detail and completed the informed consent process. Research assistants arranged for some to be interviewed in private locations in the health facility, others at their homes at a later time. Written informed consent for the interview and for being audio-recorded was obtained from all study participants prior to the interview.

In studies with non-probabilistic sampling such as this one, saturation, defined as the point at which no new themes or information emerges during the data collection, is a commonly used threshold for sample size [29]. By the end of the interview process we had reached saturation regarding the main topic of interest: the feelings experienced and the description of the types of social support that were helpful and hurtful.

### Data analysis and management

All tape recordings were transcribed; codes were then developed based on the topics of interest that emerged from the transcripts. Transcripts were then re-read and coded and analyzed both manually and using ATLAS.ti [30]. We present the results associated with social support that emerged, using representative quotes when appropriate.

### Ethical conduct of research

Prior to initiating this study, approval was obtained from the Institutional Review Boards of Tulane University School of Public Health and Tropical Medicine and from three local review boards: Peru’s Instituto Nacional de Salud del Niño, DISA-Sur authorities, and the Asociación Benéfica PRISMA (a local non-governmental organization).

## Results

Overall, participants report that social support from their family members and healthcare providers was crucial toward their psychosocial wellbeing and motivated them to adhere to their treatment; despite this, many patients described feeling depressed or even suicidal because of their diagnosis. Due to stigma related to TB in the community, few patients revealed their diagnosis outside their family or closest friends. Regarding programmatic improvements for the TB program, patients recommended improving health education for patients and family, expanding specialized support to children co-infected with HIV, and offering additional economic support in the form of food or job assistance to affected patients.

### Disclosure of disease status

Respondents tended to be very selective about which people in their lives they would tell that they or their children are TB or TB/HIV positive. Many participants described a fear of being isolated when diagnosed and expected that others might avoid them after hearing about their diagnosis, and as a result, some chose not to tell their friends or family members. Over half of respondents limited their disclosure to a close circle of entrusted family, and very few respondents (4/43) showed willingness to disclose to members of the community at large such as teachers, neighbors, or colleagues (See Table 1).

**Table 1:** Disclosure of TB and TB/HIV positive patients

	**TB adult (n = 19)**	**TB/HIV adult (n = 8)**	**TB child (n = 13)**	**TB/HIV child (n = 3)**	**Total (%)**
*Told nobody*	--	--	1	1	**4.7%**
*Only family*	8	5	7	2	**51.2%**
*Family* &*friends*	8	3	2	--	**30.2%**
*Other community (work, neighbor, teacher, etc.)*	2	--	2	--	**9.3%**
*No information	1	--	1	--	**4.7%**

*The question was not addressed in the interview.

### Role of family

Despite fears regarding alienation or isolation from loved ones, all but two participants did disclose their disease status to all or part of their families, and in response, the majority received abundant love, encouragement, financial assistance, and other forms of psychosocial support. About half of all participants discussed the value of the emotional support provided by their families, mainly in the form of encouragement that lifted their spirits, and in several cases, gave them a “reason to live”. One man with TB/HIV describes how his mother would encourage him to exercise and eat, and adds: “This is what made me get up each day.”

Family members reminded patients to go to the clinic to take their pills and often accompanied them there. Since the nausea and fatigue associated with the medication tended to make patients lose their appetite, family members countered this by cooking for them or making their favorite dishes. When comparing the amount of family support received by gender, men—particularly single men—talked about receiving psychosocial support from their families more frequently than females did.

Numerous participants also talked about the economic assistance they received from their families, and in fewer cases, from friends. Although TB patients receive medications and tests for free through the Peruvian TB Program, they described certain tests and medical-related costs that were not covered. Some study participants had to stop working or work much less, and this, combined with the additional costs associated to the disease, would have been difficult to bear without their family’s support, which came in the form of childcare, transportation, and food.

A few isolated cases received negative reactions from family members. Two TB/HIV positive participants described feeling hurt by comments from family members insinuating they must have had sex with other men or sex workers to contract this disease. Another patient, who was in treatment for TB with her child, described her sister’s distancing words and actions: reminding other family members they were contagious and to avoid sharing eating utensils with them, and not letting her nephews and nieces play with the patient's child. Oftentimes, however, family networks were extended and multi-generational, and if certain members of the family reacted negatively, others would still offer support.

Regarding relationships between partners and spouses, almost all adult patients with TB expressed receiving consistently positive support from their partners, with significantly more females than males noting the importance of the psychosocial support they received from their partners. There was a marked difference in the number of break-ups observed among participants who were co-infected with TB/HIV and mothers of children with TB/HIV compared to those who were only diagnosed with TB. Several of those co-infected stated that learning of their HIV diagnosis led to the termination of their relationships. During confrontation with their partners about their HIV status, some of the co-infected learned they had been misled or lied to by partners who knew they were infected with HIV, but had not told them, leading to their becoming seropositive and in some cases, transmitting HIV to their children.

### Role of healthcare providers

The vast majority of participants described very supportive interactions with their health care providers, who included doctors, nurses, health promoters, psychologists or nutritionists. These interactions ranged from encouraging words (e.g., encouragement to eat or to keep taking medications), to being treated with compassion and affection. Patients appreciated that healthcare providers—nurses particularly—would give hugs, make jokes, and were not “afraid” to touch them or speak at close range to them. Many of the participants described how this warmth and care—often in the form of a big smile—motivated them to continue treatment since they looked forward to seeing a cheerful nurse or, as in the case of one participant who had not told her friends and family, someone to talk to. Nurses tended to be known most familiarly by patients, and were often described affectionately and by name in the interviews.

Additionally, participants noted the importance of feeling that the provider understood their perspective, including their fears and concerns; not surprisingly, this sense of being understood led participants to open up more with their providers, as the following words of a young female participant with TB illustrate:

“So Rosa^a^, who is a very happy nurse, youthful, gives you the feeling you can ask her anything. Doctor Sanchez^a^ is a more serious doctor who I do not think cares as much about her patients as Rosa. With Doctor Sanchez it was like you go, take your pills, and that’s that. But with Rosa … you can stay hours asking her questions, talking to her, she asks you how you have been; it is like that always with her. There is a constant concern from her… Doctor Sanchez would attend you and then tell you goodbye. Instead Rosa, she gets more involved in your life. The patients are more important to her…”

However, a handful of patients also spoke of interactions with providers that were hurtful and demoralizing. Within our sample these comments happened to all be directed specifically at doctors (as opposed to nurses or other healthcare workers) who were described as “too serious” or “cold” to the point that some patients became uncomfortable or frightened to return for further consultations, or in the case of one patient, she did not take her family for sputum samples as requested by the doctor, in fear they would be “screamed at” like she had been by her doctor. Patients who expressed being fearful or intimidated by their doctors usually were able to switch doctors and found someone who was a better match for them, and in fewer cases patients sought care at a different facility.

In addition to providing key psychosocial support and encouragement, healthcare providers also played a crucial role in educating patients about what causes TB infection and the details of the treatment process. About half of the participants appreciated receiving information from their providers about their disease and what they could expect at different stages of their treatment. Clinics hosted regular health education seminars for patients experiencing TB infection, which appeared to be well received and well-attended by participants; some patients repeatedly mentioned that this information was a significant component of their treatment and recovery.

### Mental health

At the time of their diagnosis, a large proportion of participants expressed feelings of depression, loneliness, and concern for what the future might bring to themselves and their families. When deciding whom to disclose their disease status to, roughly one third of participants expressed a fear of abandonment from family members, friends, and partners. Some had become isolated within their existing social support networks, particularly those who felt they could not talk openly about their illness with family and friends. Some, advised by doctors not to work while on treatment, expressed feelings of anxiety related to their lack of income during that time.

Numerous participants highlighted that upon receiving their diagnosis as TB or TB/HIV positive, they were not aware of the availability of free treatment, and thus initially thought that they would die from their illness. Others were psychologically overwhelmed by the many life changes that were necessary for successful recovery: the feeling that they were becoming a burden to their families, the need for frequent trips to the neighborhood clinic to take pills, and suffering the debilitating affects of their medications. Four of the participants expressed that they considered taking their own lives after receiving their TB diagnosis. As one 24-year-old male describes: “I even thought about killing myself; the idea passed through my head. Thank God I got treatment from the psychologist and that helped a lot… Every time I saw someone, I thought it would be the last time I would see them."

Many participants expressed the desire to see the psychologist more often. They stated that the program allowed them one visit with the psychologist at the beginning of their treatment, with one monthly visit thereafter, but that this was not sufficient. The poverty of this allowance seems quite obvious: emotions vary as one goes through the treatment.

Parents of pediatric TB and TB/HIV patients were often infected themselves with TB and/or HIV, and thus were confronted with the double burden of providing care for their children while simultaneously coping with the physical and psychological aspects of their own illness. Some mothers expressed feeling guilty for their children’s illness; in the case of HIV, for having infected their child though vertical transmission; in the case of TB, for not having fed their child well enough to avoid active TB infection.

### Evolving social networks

Our findings indicate that the diagnosis and treatment of the participants’ illness often changed their social habits. For a variety of reasons, roughly one third of participants reported that they had increasingly withdrawn from their existing social networks since their diagnosis. Some participants had made an active decision not to socialize frequently out of fear of infecting others, or fear that members in their community would notice that they appeared ill. Others mentioned that the medications made them feel so queasy and tired that they no longer felt like going out in public. A number of TB positive men, for example, mentioned that they used to play soccer and then drink beer with their friends, but during treatment had no energy for soccer and were not allowed to drink alcohol, so as a result they saw their friends significantly less.

Despite participants’ description of increased social isolation, an overwhelming majority of adult TB patients expressed having made valuable new friendships with other patients in their treatment program with whom they could discuss their illness openly and feel supported by. Although structured social support was not available for TB patients through the clinics, participants noted that they regularly saw their fellow patients when coming to the clinic to take their pills or to attend a health education session, and bonded with them through their interactions at the clinic. Parents of pediatric patients described meeting other parents at the health education sessions and striking up mutually supportive friendships. In addition, the healthcare personnel, particularly nurses, were often described as having become important new friends in the eyes of patients. When one 18-year-old TB positive female was asked whether she had made any new friends since her diagnosis she replied:

“Yes, everyone in the program, we are already friends. Everyone—the doctors, the nurses, and everyone that is around the clinic—they greet me, ask me how I’m doing, they tell me that I look healthier, so I think we are all friends; everyone who works, and everyone that is in treatment…”

In the case of patients with TB/HIV co-infection, the organized opportunities for patients to receive social support are even more plentiful. There are health facility sponsored support groups and several described new friends whom they met during the group time, as well as at other times including weekends to socialize. In contrast, there are no facility sponsored support groups for TB patients, which was something that several TB patients noted that they would like to have available to them. They noted that having a space where patients can express their feelings and struggles would be beneficial to all to have an outlet to socialize and feel they are “not alone” by organizing social events.

### Perceived stigma

Nearly all participants acknowledged the existence of negative stigma surrounding TB and TB/HIV infection present in the community. The perceived stigma manifested itself in a variety of forms, but most commonly, participants expressed feelings of embarrassment of having an illness that is highly contagious, fearing that people will want to distance themselves. Despite the fact that drug-susceptible TB patients who have taken appropriate TB medications for six weeks or more are virtually non-contagious [14], there was a strong stigma attached to being treated for TB. Many participants expressed that they were initially embarrassed to be seen going to the clinic to take TB medication, and some sought out treatment at a facility that was located further from their home in order to avoid being seen. Mothers of pediatric patients consistently described a desire to protect their children from the stigma they might encounter from peers or the wider community by only disclosing to a select group of trusted friends or family. Only 2 out of 13 mothers of pediatric TB patients informed the children’s school that their child was infected with TB. Moreover, about half of the mothers described guilt about their child’s TB diagnosis, feeling that insufficient care or nutrition had led to their child’s illness. Multiple participants commented that TB is often referred to as a “sickness of the poor” and that many people in the community who are not well educated about TB perpetuate this and other negative stereotypes. One example of stigma in the community came from the mother of a child who has TB. She described feeling stigmatized by her own church Bishop, who upon finding out that her child was infected with TB, banished them both from attending church until the child was healthy again.

When those co-infected with TB and HIV were asked to compare the extent of stigma that exists around TB versus HIV, they felt the stigma for HIV patients is somewhat stronger. Some mentioned their perception of this increased stigma for HIV versus TB is in part that HIV is more closely associated with death, and others explained HIV’s implicit association with homosexuality in the community. A 33-year-old HIV positive man explains: “With HIV it’s different, people in the street start to make mean comments about you that are out of context; they call you a faggot. This is why I prefer not to tell other people.”

Within the clinical setting, participants noted that longer wait times might deter those patients who are embarrassed or scared that someone will see them from continuing treatment: one young man describes how he would arrive daily to the clinic, look around for anyone he might know before going to the waiting area, and if he did see someone who knew him, how he would hide elsewhere until no one might recognize him. Participants also complained about the lack of privacy. As one 22-year-old male with TB describes, “There should be a room, not an exposed [open] room where people can see you, because it is always embarrassing. More than 90% will tell you that they are embarrassed to take their pills when everyone can see them.”

### Participants’ Recommendations to improve TB programs

Participants mentioned improvements needed in the following areas: 1) Health education for patients; 2) Special needs of children with TB and TB/HIV; 3) Health education for patient families and community; and 4) Additional programmatic support.

#### Health education for patients

Several participants suggested that it would be more effective to provide information to patients in relation to their phase of treatment. One 18-year-old adult woman with TB explained:

“Right now the health posts have it wrong. When I go to pick up my food basket, they talk to us about different TB concerns. We are about 15 people, and several of us are about to finish treatment. So they give us talks that they should have given us at the start of treatment, like what tuberculosis is. They should separate us into groups. Those who are just starting should be separate from those of us who are finishing treatment.”

Related to this, several participants suggested asking people who had previously been infected with TB to work as health educators so that they could build positive relationships with patients and teach them from their own experiences, i.e., peer educators. One 18-year-old woman in treatment for TB said: “The former patient knows what the patient feels like. They know better than anyone: better than the doctor, better than all.”

#### Special needs of children with TB and TB/HIV

The parents of children with TB and TB/HIV expressed a desire to receive more specialized education, counseling and advice with regard to specifically caring for their children. Additionally, parents want health providers who have certain qualities important for working with children, such as a personable approach (greeting children with a smile), having extra patience, making children feel comfortable and providing a peaceful ambiance. One parent of a child with TB/HIV recommended that “they should treat a child with affection, that’s most important, treating them well, showing them that they will get better, smiling at them.” Some parents felt that a separate facility just for children would be ideal. Other parents expressed a need for more economic assistance, more home visits and experienced doctors rather than student residents to provide these services.

#### Health education for patients’ families and community

Numerous participants noted the need for increased TB education to the general public and patients’ families, such as TB campaigns in the media, programs in schools and outreach to families. A parent of a child with TB noted, “If I could, I would show a program on the TV or radio… so that parents can learn what to talk to their children about if they have this disease and to know how to prevent this disease.” One 18-year-old female with TB suggested “community campaigns to teach people what it is, how it is cured, how to avoid it…” Several participants stated that educating the youth through their schools could be an effective vehicle for reaching large numbers of people, but that the education of TB and other illnesses must be thorough.

The participants noted the need of specific education for families and friends of patients so that they can better understand the dynamics of treatment and the illness. One adult male with TB stated the need for “explaining at the very least to the people closest to me what they need to do to be careful.” Another adult male with TB/HIV recommended “inviting the family member of a patient who does not consistently attend his or her medical appointments to serve as a support for that patient.”

#### Additional programmatic support

Other recommendations included the provision of breakfast for people when they come in the morning to take their medications, more variety in the food given to the patients as part of the National TB Program, for food to be given consistently each month, an increase in the number of home visits, the availability of all services at the same location, the ability to take medication home if the facility schedule interferes with work, free consultations with doctors when needed and a vocational training program. A 33-year-old male with TB/HIV noted that the National TB Program could do more to help those who can’t find work: “There are many people who don’t have work; provide a workshop, to help us in some type of way. It could be to learn to knit sweaters, make shoes, carpentry - these skills can help people defend themselves, because for those with this illness, sometimes people at their work find out and they are fired and they are left with nothing.”

## Discussion

The provision of unique forms of social support (or lack thereof) from family, caregivers, and the wider community plays a distinct role in affecting patients’ psychosocial wellbeing. Outside factors such as poverty, add layers of complexity to patient psychosocial needs, and thus the types of programmatic interventions and future research needed to adequately address them.

### Social Support: the role of family, friends & community

Compared to other social groups, families were the most highly involved group in terms of supporting and caring for patients as a part of their daily routines; providing regular encouragement for patients to eat, take their medications, and maintain a positive outlook. It is interesting to note that the majority of participants who described the emotional support and caregiving received from their family as the most important type of support were single men, likely receiving it from their mothers and sisters. Studies on TB and gender denote that women are often more caring of other members of the family and continue in the role of caretaker even when sick. When men and children in the home are sick, women will prioritize their needs to provide better care for them [25,31]. Another study from Peru found that the burden of caring for TB patients tended to fall on women [32]. A future study that incorporates interviews with contact cases of TB patients would be an interesting way to understand the caregiving role of women and how it evolves if they contract the disease themselves.

Regarding partner support, more women than men expressed the significance of their partners’ involvement; three-fourths of those who mentioned partner support were women. Research affirms that women may express more importance in their husband’s presence and fears of him leaving them during the sickness because of economic dependence [22,25]. It may also be that stereotypically women are expected to provide this type of support and thus it is taken for granted by some men. Health practices must address partner issues that arise during TB treatment, such as sleeping arrangements while patient is contagious, and relationship breaks-ups as a result of disclosure. Further research would be useful in order to understand what actions partners of TB patients should take to optimally support the psychological and physical needs of their partner.

In examining the role of social support from the wider community, we note the enormous potential for members of the community to be a valuable source of social support to patients. The majority of participants did not tell community members about their sickness due to fear of discrimination or embarrassment, as has also been found in other studies [33-36]. Reactions like those of the Bishop banishing the woman from church contribute to the fears of many patients with TB, who then do not tell people beyond their closest network of family and friends. However, contrary to what many participants expected, this study found that most of those who disclosed their status to a wider audience received positive support and made new friendships as a result of their disclosure. If structured support groups or associations were available for TB patients, similar to those available for HIV patients, they could facilitate supportive interactions among TB patients and improve patient psychosocial wellbeing. Research in other developing countries has demonstrated that social support groups such as “TB clubs” have facilitated a greater understanding of the disease, positive relationships with health personnel, and a more positive attitude within the community, resulting in less stigmatization and more community support [23].

An interesting and unexpected observation from this study was that patients’ social networks tended to evolve (vs. diminish or expand) when comparing social habits and friend groups pre- and post-diagnosis. Roughly one third of participants reported feeling increased social withdrawal from their existing social networks after their diagnosis. Some explained that they felt less sociable because they were afraid of infecting others or of being judged by their peers, while others simply felt too physically ill from their treatment to engage in their regular social activities. Given the apparent mental health needs within this patient population, it is likely that feelings of depression also contributed to social withdrawal. A woman positive for TB and HIV attributed her initial social withdrawal to the fact that she was jealous of her “healthy friends”, then subsequently made new, supportive friends who she met while taking TB medications at the clinic. In the case of this woman, and the other participants who similarly left behind their old social networks while they focused on their treatment, it would be interesting to know what long-term implications this might have on their social life and their psychosocial wellbeing. Will they rejoin their old friend group eventually? Likewise, for those who made new friends during treatment, will they likely keep those friendships in the long-term after they are cured?

### Importance of positive patient-provider interactions

The significance of the patient-healthcare provider relationship and its influence on the patient’s desire to return to the health facility or not, as well as the patient’s trust in their health provider, was a key element in the results of this study, as has been found in others [22,37]. Certain components of patient-provider communication, in particular a caregiver’s ability to listen and show respect to their patient, are important determinants of the patient’s healthcare utilization [38]. Since many patients exhibited low self-esteem and feelings of social isolation, the kind words and generally supportive attitude of the healthcare staff were important factors in encouraging participants to continue with their treatment in spite of the physical and psychological difficulties associated with the treatment process. Given the particular importance of treatment adherence among TB patients in order to avoid developing more dangerous drug resistant strains of TB, it is important that stakeholders in the TB community understand the potential utility of well-trained healthcare providers. Regular training and refresher workshops for healthcare providers that address emotional support and bedside manner (patient and health provider interactions), including basic communication skills, could be helpful, as requested by the study participants.

The level of healthcare staff motivation and the quality of their interactions with TB patients is one of seven types of interventions intended to improve adherence that is currently being evaluated by the Cochrane Collaboration [3]. The majority of negative interactions described between patients and healthcare providers involved doctors who seemed to use fear and intimidation tactics to scare patients into treatment compliance. What we found was that those providers who used these methods to motivate participants to return usually only discouraged them or led the participants to seek out other providers or, in a few cases, other facilities for their care. However, our limited sample focused on individuals who were in treatment, so we did not capture patients who had negative treatment outcomes nor the reasons they had for discontinuing treatment.

### TB & poverty: further economic support for this impoverished population

The need for further economic support from families, friends and the community also emerged as a recurring theme from participants in this study. It is inevitable to discuss the issue of poverty when writing about TB. Poverty is a risk factor in becoming infected with TB, and results demonstrate that already impoverished families become further burdened by this stress when dealing with TB [4,5,22,26,39]. Although Peru’s TB program provides a food basket and free medications to TB patients of low socioeconomic status, participants indicated that they would appreciate further nutritional support, such as the provision of breakfast at the clinic when coming to take their pills in the morning. Further economic hindrances to completing treatment were described, such as transportation costs, and costs for required additional medical treatment, such as X-rays and blood work or care from other specialized physicians. Some of these services should be covered, but these are difficult for some patients to access, which results in patients paying for them. Because a subset of the study participants was also co-infected with HIV, it is worth noting that these patients represent a particularly economically vulnerable group. TB/HIV patients cope with a lifelong condition that forces them to manage multiple diseases and/or opportunistic infections, often having to seek treatment at multiple facilities within one day, which can inhibit their ability to generate income over their lifetime.

Another related issue is that since many TB patients must leave their jobs during treatment, some get fired as a result. This loss of income can be devastating not only for the patient but also to the family that they had previously been supporting. The incorporation of group income generating activities into the current TB program, as was suggested by participants, could be a productive way to both encourage socialization among patients and contribute to their economic wellbeing.

### Depression is common: staged mental health interventions needed

An important observation from this study for its programmatic implications was the extent of apparent mental health needs among the patient population. Participants consistently expressed the importance of their monthly psychological consultations in helping them “keep going” and their desire for the visits to be more frequent. Within our sample of 43 participants, there were 4 patients who had considered taking their own life as a result of their diagnosis. Psychological issues associated with TB diagnosis have been shown to manifest as a result of patients’ feelings of fear and guilt of infecting others; social discrimination, the socio-economic stress and psychological burdens of living with a life-threatening illness; increased dependence on others; and concomitant poverty [40]. A 2007 mental health assessment of rural Lima shows that about 20% of the inhabitants had ever felt either moderately or severely depressed, citing poverty and unemployment as the main areas of preoccupation for this population—no literature was found on depression rates in peri-urban locations [41]. Several other authors have described how psychosocial factors such as anxiety and depression can complicate adherence to drug regimens, and emphasize the importance of attention to mental health needs to ensure positive treatment outcomes [40]. The mental health needs of these patient populations are numerous, relevant in terms of treatment outcomes, and perhaps not being met to the fullest extent.

In this study patient mental health needs appeared to be highest during the period immediately following their diagnosis of TB or TB/HIV. During this period, patients faced a barrage of emotions including shock, embarrassment, and concern for their families. Also during this initial phase, patients did not have the opportunity to receive education regarding what to expect in the treatment of their illness and are particularly fearful of what their future might hold. This period should be recognized as a critical time for educational and psychological intervention to ensure that patients feel supported to move forward with their treatment. Again, it is important to note that our sample consisted only of patients in treatment (the “success” cases); it would be interesting to know what proportion of patients who left treatment could have benefitted from enhanced psychosocial support following their diagnosis. Programmatically relevant, those at later stages of treatment commented the need for time-relevant psychological support: patients at different stages of treatment should not be receiving the same counseling.

Interviews with parents of pediatric patients revealed the difficulties faced by parents who were also infected with HIV and/or TB in providing care and support for their children. All mothers of pediatric patients positive for TB and HIV were HIV positive themselves, and some parents of TB patients were also being treated for TB alongside their children. Whilst these parents experienced fatigue and nausea from their own treatment, they also were forced to find the strength and motivation to care for their children; feeding them, taking them to the clinic, and consoling them. Moreover, they communicated their efforts to hide their true emotions of sadness, frustration, or guilt in order to protect their children from feeling abnormal or stigmatized.

### Patient requested programmatic activities: peer support and educational opportunities

Several participants expressed a desire for increasing supportive peer education activities, directed at patients, to be incorporated into the existing TB program. Peer support models have indeed been proven as an effective tool to influence patients’ health-seeking behaviors and serve as a complement to clinical services, particularly in the field of HIV education and prevention activities [42]. Similar to the case of HIV, TB is also an illness that is clinically complex, manifests itself in various stages, and is often attached to social stigma, making the need for patient education and psychosocial support crucial. The value in peer support is that it empowers patients to participate in their own healthcare rather than relying on healthcare professionals to enforce treatment compliance. There has been little published on the potential for using peer assistance interventions to improve TB patient education and encouraging medication adherence. However based on their success in the context of HIV, as well as the request from our study participants, the use of peer educators could prove to be a valuable and cost effective way to supplement clinical care TB in resource-constrained settings.

Finally, as a recommendation to improve the type of support and understanding participants wanted from others, most suggested community-wide TB and HIV education programs, tailored for various audiences, including patients themselves, families, partners and the wider community. Research demonstrates that receiving more information on the disease as a patient empowers the patient to feel more confident and active in his or her treatment. Likewise, when families and support networks are well informed about the treatment process, patients experience less social isolation [37]. That said, future research on this topic could focus on questions like: what role can families play in encouraging patient treatment adherence? Will patients and families participate in support groups or will these be avoided due to possible stigma?

### Limitations

Our findings capture certain issues and trends as observed by a small group of people being treated for TB or TB/HIV, but these are not representative of the general population, nor are they meant to be. All of the patients interviewed were recruited through a health care provider, were currently in treatment for TB, and were willing to speak with someone regarding their specific health situation; there is a clear selection bias. Also, since participants selected had been in TB treatment for at least a few weeks, so that we could obtain a broader perspective on social support issues throughout the course of their treatment, there could be an issue of recall bias regarding emotions or social support at the earlier phases of treatment. Although the recruitment process for pediatric patients targeted patients under the age of 12, the exact age of pediatric patients whose parents were interviewed was not recorded. Due to our sampling design, we did not speak with any patients who had stopped receiving treatment, yet this would be a particularly important group of people to interview in future research. Finally, the focus of this research was on social support during treatment for TB, and though we included individuals with HIV co-infection, we did not probe on specifics about people’s HIV treatment or managing their health care needs associated to both diseases—yet there is obviously much more to be learned from this sub-group. We also did not find out about the stage of their HIV disease, which is particularly relevant since we observed evolving issues related to their mental health and social support after diagnosis with TB, and would expect similar changes at different stages of HIV disease.

## Conclusion

Various types of support play a significant role in patients’ recovery and treatment outcomes, and although the family is a key social support provider, it is clearly not the only support system that can be tapped into. Families, friends, and healthcare providers all contributed to the patients’ support system in different ways, but as expressed by patients themselves, educational programs targeting these different groups would enable them to better support the patients. Patient-provider interactions were described as overwhelmingly positive, which likely contributed to improved patient psychosocial wellbeing. Perceived stigma in the community, lack of psychosocial support, and forced lifestyle changes associated with TB and TB/HIV positive diagnosis contribute to the significant mental health needs of this patient population. Unmet need for psychological support to help patients cope with anxiety, depression, and frustration associated with their TB diagnosis may cause a reduction in patient quality of life and lead to decreased treatment adherence. To meet patient psychosocial needs and to improve the Peru TB program overall, participants made valuable suggestions which should be considered in future development of TB support programs: extending disease-specific educational opportunities to patients’ families and the wider community; increasing the existing amount of nutritional support; program provision of vocational activities to increased economic opportunities; and increased frequency of psychological counseling. Future research on the topics of the evolving social networks of TB patients, links between mental health needs and treatment adherence levels, staged implementation of patient psychological support, and the quantification of TB stigma would be useful contributions to this field. Bridging the gaps that remain among communities, families, patients and health providers through training, health education and improved communication could ensure a healthier, more informed population that is better equipped to provide social support to individuals who need it.

## Endnote

^a^Names have been changed to protect privacy.

## Competing interests

The authors declare that they have no competing interests.

## Authors’ contributions

VPS conceived the study, managed data collection, analyzed data, wrote and edited the article; RA analyzed data, wrote and edited the article; CD analyzed data, wrote, and edited article; RO conceived study, wrote and edited the article. All authors read and approved the final manuscript.

## Pre-publication history

The pre-publication history for this paper can be accessed here:

http://www.biomedcentral.com/1472-6963/13/290/prepub
